# CD133 Expression Is Not Synonymous to Immunoreactivity for AC133 and Fluctuates throughout the Cell Cycle in Glioma Stem-Like Cells

**DOI:** 10.1371/journal.pone.0130519

**Published:** 2015-06-18

**Authors:** Alonso Barrantes-Freer, Mirjam Renovanz, Marcus Eich, Alina Braukmann, Bettina Sprang, Pavel Spirin, Luis A. Pardo, Alf Giese, Ella L. Kim

**Affiliations:** 1 Molecular Biology of Neuronal Signals, Max-Planck-Institute of Experimental Medicine, Göttingen, Germany; 2 Institute of Neuropathology, University Medical Centre, Göttingen, Germany; 3 Translational Neurooncology Research Group, Department of Neurosurgery, Johannes Gutenberg University Medical Centre, Mainz, Germany; 4 Institute of Toxicology, Johannes Gutenberg University Medical Centre, Mainz, Germany; 5 Engelhardt Institute of Molecular Biology, Russian Academy of Sciences, Moscow, Russia; 6 Translational Neurooncology Research Group, Department of Neurosurgery, University Medical Centre, Göttingen, Germany; University of Florida, UNITED STATES

## Abstract

A transmembrane protein CD133 has been implicated as a marker of stem-like glioma cells and predictor for therapeutic response in malignant brain tumours. CD133 expression is commonly evaluated by using antibodies specific for the AC133 epitope located in one of the extracellular domains of membrane-bound CD133. There is conflicting evidence regarding the significance of the AC133 epitope as a marker for identifying stem-like glioma cells and predicting the degree of malignancy in glioma cells. The reasons for discrepant results between different studies addressing the role of CD133/AC133 in gliomas are unclear. A possible source for controversies about CD133/AC133 is the widespread assumption that expression patterns of the AC133 epitope reflect linearly those of the CD133 protein. Consequently, the readouts from AC133 assessments are often interpreted in terms of the CD133 protein. The purpose of this study is to determine whether and to what extent do the readouts obtained with anti-AC133 antibody correspond to the level of CD133 protein expressed in stem-like glioma cells. Our study reveals for the first time that CD133 expressed on the surface of glioma cells is poorly immunoreactive for AC133. Furthermore, we provide evidence that the level of CD133 occupancy on the surface of glioma cells fluctuates during the cell cycle. Our results offer a new explanation for numerous inconsistencies regarding the biological and clinical significance of CD133/AC133 in human gliomas and call for caution in interpreting the lack or presence of AC133 epitope in glioma cells.

## Introduction

A transmembrane protein CD133 (also known as prominin-1) is expressed in hematopoietic and neural stem cells (NSCs) [1, 2] and glioma stem-like cells (GSCs) [3, 4]. In GCSs, certain patterns of CD133 segregation are associated with distinct modes of cell division implying a role of CD133 in maintaining the pool of GSCs and regulating their cell fate [5]. Several lines of evidence suggest a link between the surface expression of CD133 and tumorigenicity of glioma cells. Indeed, several studies found that glioma cells immunopositive for the AC133 epitope located in the N-terminal extracellular loop of CD133 manifest greater malignant potential when compared to their AC133-negative (AC133-) counterparts from the same tumor [3, 4, 6]. Further evidence for the link between CD133 and tumorigenic capacity of GSCs comes from studies showing that CD133 knock-down impairs GSCs self-renewal and tumorigenicity [7, 8]. Furthermore, numerous studies have reported on the direct correlation between CD133/AC133 expression and poor clinical outcome in patients with GBM [3, 4, 6, 9–14]. However, there is also an increasing number of studies that have questioned the significance of CD133/AC133 as a potential biomarker of GSCs [15–23] or positive predictor of GBM aggressiveness [16, 18, 24, 25]. Moreover, one study, in which evaluation of CD133 levels was independent from the AC133 epitope, found an inverse correlation between CD133 expression and GBM aggressiveness [16]. The reasons for these discrepant results remain unclear and the need for clarification of existing controversies has been repeatedly emphasized [26–29].

CD133 expression in glioma cells is commonly evaluated by assessing the expression of cell-surface epitope AC133. It is widely assumed that the level of AC133 expression reflects the level of CD133 protein in glioma cells. Another key assumption that had been taken for granted is that CD133/AC133 positivity of glioma cells marks a specific cell fate, namely that of undifferentiated GSCs. However, neither of these widespread assumptions has been tested rigorously in glioma cells. In this regard, it should be noted that previous studies in glioma cells did not take into account that lack of AC133 immunopositivity may not always be synonymous to the lack of CD133 protein as shown in other cell types [30–32]. Furthermore, it is known that accessibility of the AC133 epitope is not permanent but a subject to modulation by various factors including structural changes in the plasma membrane during differentiation, alterations in the glycosylation processing [33, 34], change in the bioenergetic status [35], or epigenetic modifications [36]. In the context of existing disparities around CD133/AC133, the importance of addressing methodological issues has also been intensely discussed in the literature, with particular emphasis on the limitations of anti-AC133 antibodies (AC133 Ab) widely used to isolate GSCs and evaluate CD133 expression in tumour specimens (rev in [27, 28, 32]. One concern is that AC133 Ab-coated microbeads that have been and continue to be used to isolate GSCs from fresh tumour specimens can bind glioma cells irrespective of CD133/AC133 expression [18]. Despite the intense debate on the reliability of the AC133-based approach, very few studies have undertaken a direct comparison between the results obtained with AC133 antibodies and those obtained from CD133 assessments independent from the presence of AC133 epitope. Underscoring the importance of validating the outcomes from studies that have used GSCs selected on the basis of their immunopositivity for AC133, the conclusion that CD133/AC133 is a marker of stemness in glioma cells was not verified when the expression of CD133 was evaluated by a combination of quantitative and qualitative techniques [37]. A further complication is the non-uniformity of sources (freshly resected tumour specimens, conventional glioma cell lines or human glioma xenografts) that have been used in different studies to isolate GSCs. Fresh tumour specimens are generally considered the most preferable source of GSCs. However, they may also contain CD133 expressing endothelial cells, which have been shown to support the tumour-propagating capacity of glioma cells [38]. Thus, it is conceivable that the presence or absence of CD133+ endothelium co-purified with CD133+ GSCs may be an important factor influencing the tumorigenicity of GSCs that have been derived directly from surgical specimens but not of GSCs isolated from more homogeneous cell populations such as established glioma cell lines, which are less likely to contain human endothelial cells. Considering the potential prognostic importance of CD133/AC133 as identity marker for GSCs and a predictor of the therapeutic response in malignant brain tumours [9], the clarification of controversial findings regarding CD133/AC133 is an issue of the biological and clinical significance. In this study, we utilized different approaches to assess the expression of surface CD133 in glioma cells and correlate CD133 levels with specific biological properties and clinically relevant characteristics attributed to GSCs.

## Materials and Methods

### Cells and antibodies

All lines of human GSCs used in this study have been described in our previous publication [39] and derive from excess tumor tissue of glioblastoma patients operated at the Department of Neurosurgery of the University Medical Centre Göttingen, with written informed consent obtained from patients for using excessive tumor tissue for research purposes. The use of tumor tissue was approved by the Institutional Review Board of the University Medical Centre Göttingen. GSCs were cultivated in NeuroBasal medium supplemented with the B27 component (Invitrogen Life technologies), fibroblast growth factor (FGF) and epidermal growth factor (EGF) (10 and 20 ng/mL, respectively, Biochrom GmbH, Merck KGaA, Germany). The tumorigenic potential was evaluated in an orthotopic mouse glioma model (NMRI, Charles River Europe). All lines used in this study gave rise to tumours with morphological criteria of GBM. The human colon carcinoma cell line CaCo-2 [40] was a kind gift from Dr. Frauke Alvez (University Medical Center, Göttingen) and was obtained from the German collection for microorganims and cell cultures (DSMZ, No. ACC169). CaCo-2 cells were maintained in RPMI 1640 medium (Gibco®/Invitrogen Life technologies) supplemented with 10% of fetal calf serum at 37°C and 5% CO_2_ in a humidified atmosphere. For flow cytometric analyses, we used a pycoerythrin-coupled antibody CD133/1 (AC133) (Miltenyi Biotec GmbH, Bergisch Gladbach, Germany) or uncoupled antibody Ab66141 that binds to the C-terminal domain of CD133 (Abcam, MA, U.S.A.). As an isotype control, mouse IgG1 (R&D Systems GmbH, Wiesbaden-Nordenstadt, Germany) was used. An uncoupled antibody CD133/1 (AC133) (Miltenyi Biotec GmbH, Bergisch Gladbach, Germany) was used in western blot analyses. For immunohistochemical assessments, antibody specific to human nestin (R&D Systems GmbH, Wiesbaden-Nordenstadt, Germany) was used accordingly to the supplier’s recommendations.

### Flow cytometry

Single cell suspensions of GSCs or CaCo-2 cells were centrifuged at 1200 x *g* for 2 minutes and re-suspended in PBS containing 2mM EDTA and FcR blocking reagent (Miltenyi Biotec GmbH, Bergisch Gladbach, Germany) at a concentration of 10^7^ cells/ml. Cells were fixed in 1% paraformaldehyde for 5 minutes at +4°C and washed three times with PBS. Blocking was done with the FcR blocking reagent (Miltenyi Biotec GmbH, Bergisch Gladbach, Germany) for 20 min at room temperature. Flow cytometric immunophenotyping was performed with fluorophore-conjugated antibodies from Miltenyi (Gladbach, Germany) following manufacturer’s instructions. For CD133 stainings, PE-conjugated antibodies AC133, CD133/2 (clone 293C3) or CD133/2 (clone AC141) were used. For CD133, CD15 and CD49f co-staining, combinations of APC-conjugated anti-AC133, VioBlue-conjugated anti-CD15 or FITC-conjugated anti-CD49f antibodies were used. For AC133/CD133CT co-staining, a mix of AC133 Ab (PE-conjugated, dilution 1:10; Miltenyi, Gladbach, Germany) and Ab66141 (unconjugated, dilution 1:100, Abcam, MA, U.S.A) was used. Cells were stained for 30 min at room temperature. After staining, the cells were retrieved by centrifugation and re-suspended in PBS. The samples were analyzed in a FACSAria flow cytometer (BD Biosciences, Heidelberg, Germany) or a FACSCanto II flow cytometer (BD Biosciences, Heidelberg, Germany) using a 405 nm, a 488 nm and a 633 nm laser for excitation. The fluorescence emission was collected using 530/30 bandpass filters for Alexa 488 and 576/26 (FACSAria) or 585/42 (FACSCanto II) bandpass filters for PE. Vioblue was detected with a 450/50, Alexa 633 and APC with a 660/20 bandpass filter. Linear forward and side scatter gates were used to eliminate cell clumps and debris. After gating, a minimum of 10^4^ events was recorded for each sample. FACS Diva v 5.0 software (BD Biosciences, Heidelberg, Germany Biosciences) was used for data acquisition and post-acquisition data processing was done with FlowJo X software (Tree Star, Oregon, USA).

### Subcellular fractionation and western blot

Isolation of plasma membranes was done as described previously [41] with some modifications. In brief, GSCs (3x10^8^) or CaCo-2 (1x10^8^) cells were washed twice in TE buffer (10 mM Tris-HCl, pH 7.6, 1 mM EDTA), pelleted by centrifugation and re-suspended in TE buffer containing 255 mM sucrose (TES). All buffers were ice-cold and contained protease inhibitors (Complete, Roche). All centrifugations were done at 4°C. Cell suspensions were incubated for 20 minutes on ice followed by ultrasonic homogenization. An aliquot of crude lysate was saved for as the input control. Cell homogenates were centrifuged at 9000 x g for 10 min. Supernatants containing plasma membranes were subjected to centrifugation at 27000 x g. The resulting pellets were re-suspended in TES and subjected to ultracentrifugation through 38.3% sucrose in TE at 100.000 x g. The interphase fractions were collected, diluted fourfold with TE buffer and centrifuged at 100.000 x g for one hour. Pellets containing plasma membranes were re-suspended in 100 μl of TE buffer and analyzed by the Bradford assay to determine protein concentration. Proteins were separated by electrophoresis through 8% SDS-polyacrylamide gels and transferred onto PVDF membranes (Invitrogen). The efficacy of protein transfer was verified by staining with Ponceau S (Sigma-Aldrich).

### RNA isolation and semiquantitative RT-PCR

RNA was isolated by using TRIzol Reagent (Life Technologies) according to manufacturer’s instructions. Oligo (dT)-primed reverse transcription of total RNA (5 μg) was conducted by using SuperScript IV First-Strand Synthesis System (Invitrogen). As a control, normal human brain RNA (Clontech Laboratories Inc.) was used. The resulting cDNA was amplified by using multiplex PCR Kit (Qiagen). The PCR primers were as follows: full length CD133 transcript, 5’-GCACGGATCCTGGAGGATCTTGCTAGCTATG-3’ (forward), 5’-GAGCTCGAGTCAATGTTGTGATGGGCTTGTC-3’ (reverse) [7]; CD133 total transcript (pos. 813–445, Genebank accession number NM_006017), 5’-TGGCAACAGCGATCAAGGAGAC-3’ (forward), 5’-TCGGGGTGGCATGCCTGTCATA-3’ (reverse) [42]; alternatively spliced transcript CD133s1 (pos. 278–431, AF507034), 5’-CAGAAGGCATATGAATCC-3’ (forward), 5’-CACCACATTTGTTACAGC-3’ (reverse) [42]; alternatively spliced transcript CD133s2 (pos. 278–458, AF027208), 5’-CAGAAGGCATATGAATCC -3’ (forward), 5’- CACCACATTTGTTACAGC-3’ (reverse) [42].

### Cell synchronization

GSCs (No. 1051) were plated in NeuroBasal+B27 medium supplemented with bFGF and EGF (NeuroBasal Complete) in T-75 flasks at a starting density of 3x10^6^ cells per flask. 24 hours after plating, thymidine was added to a final concentration of 2 mM. After 18 hours of thymidine treatment cells were harvested by centrifugation (900 x g, 10 min, 4°C), washed twice with PBS and put into thymidine-free NeuroBasal Complete medium for another 24 hours. After the second round of thymidine treatment, one half of the treated cells was directly stained for CD133 and analyzed by flow cytometry. The other half was washed twice with PBS and incubated in thymidine-free NeuroBasal Complete medium for additional 12 hours prior staining.

### Animal experiments and immunohistochemistry

The potential to initiate tumour growth was confirmed for each line used in the study in an orthotopic mouse model of glioma. Animal experiments were approved by the State Office of Lower Saxony (permission #33.942502-04/012/07) and State Office of chemical investigations of Rhineland-Palatinate (permission #23 177-07/G12-1-020). The protocol for animal experiments was approved by the Central Animal Research Facility (ZTE) of the University Medical Centre of Göttingen and Translational Animal Research Center (TARC) of the Johannes Gutenberg University Medical Centre of Mainz. Animal experiments were performed in accordance with the guidelines of the European Convention for the Protection of Vertebrates Used for Scientific Purposes. Female NMRI mice (5–6 weeks old, Charles River U.S.A.) were maintained at no more than five mice per individually ventilated cage on a 12 hour light/dark schedule at a constant temperature of 28°C and at 50% relative humidity. Cages and bedding were autoclaved and changed twice per week. For intracranial implantation, single cell suspensions were prepared from gliomasphere cultures by using a combined trypsin/mechanical trituration procedure. Cells were washed twice in PBS and re-suspended in PBS at 10^5^ cells/μl. Cells viability was determined by trypan blue staining. Prior to implantation, mice were anaesthetized by an intra-peritoneal injection of avertine at 0.4 g/kg body weight. For implantation, the cranium was fixed in a stereotactic frame (TSE Systems, Bad Homburg, Germany). 10^5^ cells were injected into the caudato-putamen of the right-brain hemisphere using the following stereotactic coordinates in reference to the bregma: 1 mm (anteroposterior axis), 3 mm (lateromedial axis), 2.5 mm (vertical axis). To ameliorate post-operative pain after intracranial implantation pain reliever (Novalgin) was added to drinking water for three days after implantation. Mice were observed daily and sacrificed at the first manifestation of neurological symptoms by injecting i.p. a lethal dose of avertine. Tumor bearing brains were explanted and fixed in 4% paraformaldehyde in PBS. After fixation, the brains were cut in coronal sections, paraffin-embedded, dissected into 1–3 μm thick sections and examined by haematoxyline/eosin or immunohistochemical staining assessments.

## Results and Discussion

**Fig 1:**
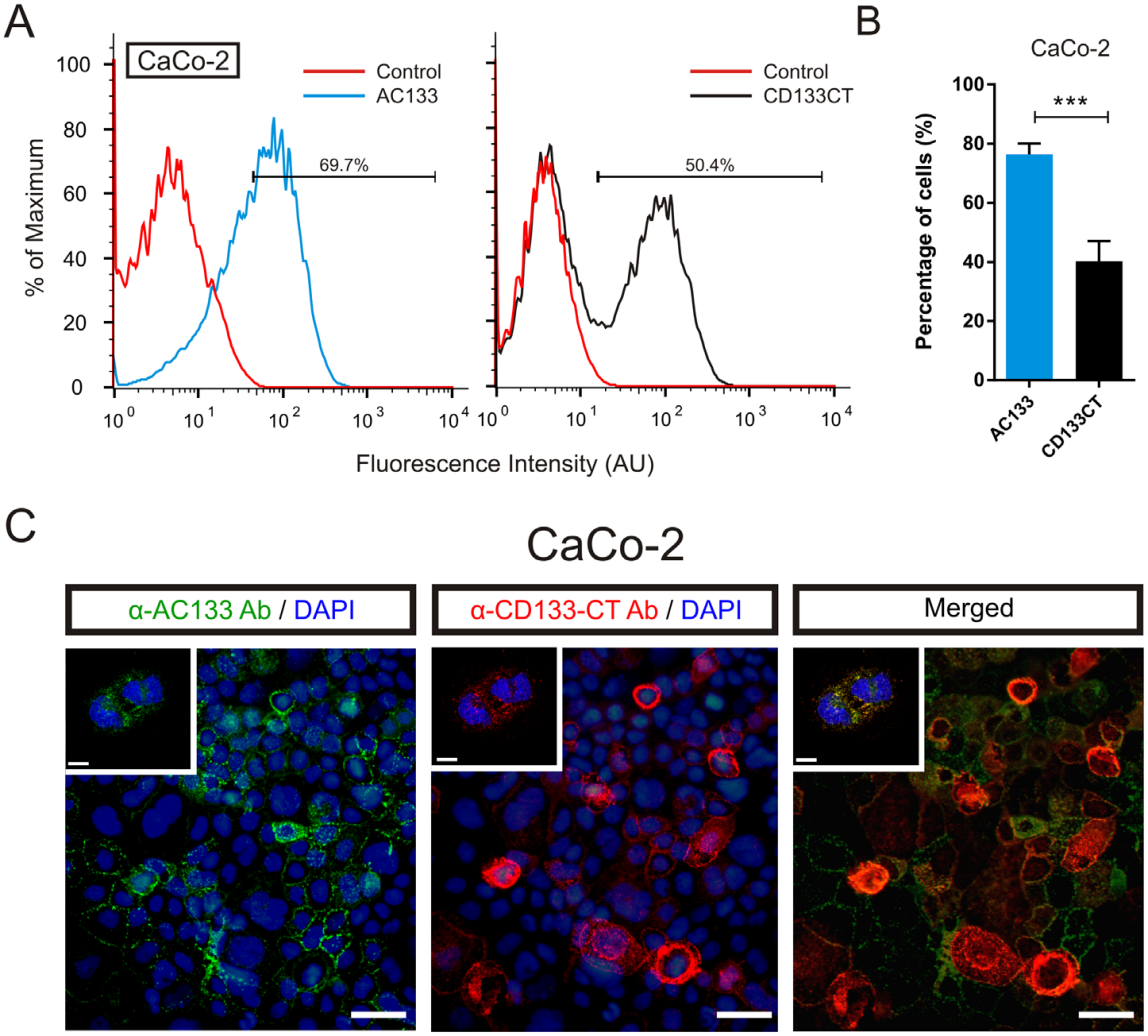
Divergent patterns detected by anti-AC133 and anti-CD133CT Abs in CaCo-2 cells. A. Representative flow cytometry histograms of CaCo-2 cells immunolabeled with anti-AC133 Ab (*left panel*) or anti-CD133CT Ab (*right panel*). B. Quantification of AC133- and CD133CT-positive cells from three independent experiments. Bars represent mean percentage of positive cells ± SEM. C. Immunofluorescence microscopy of CaCo-2 cells co-stained with anti-AC133 Ab (green) and anti-CD133CT Ab (red). Nuclei were counterstained using DAPI (blue). Confocal microscopy images (*inset*) show the subcellular distribution of the staining. Merged image shows overlapping signals generated by anti-AC133 Ab (*green*) and anti-CD133CT Ab (*red*) antibodies. Scale bars correspond to 50 μm (main fields) and 7 μm (insets).

To assess surface CD133 by flow cytometry, we have used anti-AC1331 (CD133/1, Miltenyi Biotec) and anti-CD133CT antibodies binding to different regions in the CD133 protein. Anti-AC1331 Ab binds to the externally located epitope AC133 whereas anti-CD133CT Ab recognizes the C-terminal domain of CD133 (CD133CT). We first analyzed CD133 binding patterns in the human colon carcinoma cell line CaCo-2, which is well characterized with respect to CD133/AC133 [43]. In agreement with previous reports on CaCo-2 cells, our assessments confirmed the expression of membrane-associated CD133 in CaCo-2 cells detected by both anti-AC133 and anti-CD133CT Abs (Fig 1A).

**Fig 2:**
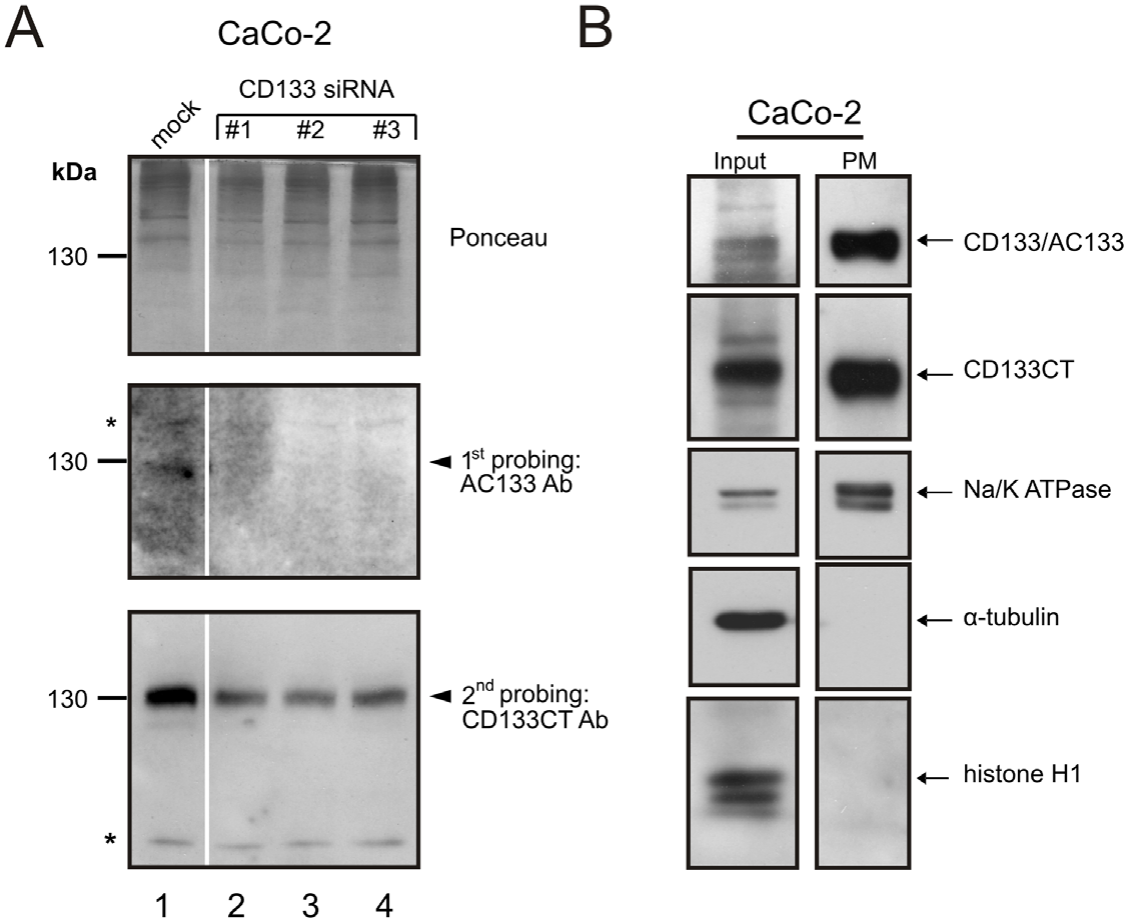
Comparative analysis of the total and membrane-associated CD133 protein in CaCo-2 cells. A. Western blot analysis of crude lysates (100 μg total protein per lane) from mock-treated CaCo-2 cells (lanes 1) or CaCo-2 cells transfected with the validated anti-CD133 siRNA (LifeTechnologies) (lanes 2–4). After the transfer to the PVDF membrane, Ponceau S staining was done to verify the efficacy of protein transfer (*top panel*) and followed by sequential probing with anti-AC133 Ab (*middle panel*) and anti-CD133CT Ab (*bottom panel*), respectively. B. Comparative assessment of CD133 in unfractionated cell lysates (“input”, 20 μg) and plasma membranes (“PM”, 1 μg) by anti-AC133 or anti-CD133CT Abs.

To detect the C-terminal CD133CT epitope located on the inner side of the cell membrane cells were subjected to a mild fixation with 1% paraformaldehyde. This condition allows for a partial permeabilization of the cell membrane thus facilitating antibodies access to the otherwise shielded intracellular epitopes of membrane-associated proteins [44, 45]. Notably, the percentage of CD133-positive CaCo-2 cells recognized by anti-AC133 Ab was consistently higher than the percentage of cells detected by anti-CD133CT Ab in flow cytometry (Fig 1B). Although immunofluorescence co-staining of CaCo-2 cells by anti-AC133 and anti-CD133CT Abs revealed overlapping staining patterns (Fig 1C, left and middle panels, respectively), not all cells stained with anti-AC133 Ab seem to be also recognized by anti-CD133CT Ab (Fig 1C, right panel). One explanation for the dissimilarity between numerical estimates obtained with anti-AC133 Ab and anti-CD133CT Ab may be a difference in accessibility of their corresponding epitopes. To test this possibility, we compared patterns of CD133 expression detected by anti-AC133 Ab or anti-CD133CT Ab under denaturing conditions, by western blot analysis. Fig 2A shows the same membrane, which was successively probed with anti-AC133 (middle panel) and anti-CD133CT (bottom panel) antibodies. The results showed that both anti-AC133 and anti-CD133CT Abs detect one common band migrating with an apparent molecular weight corresponding to CD133 (lane 1, band indicated by arrowheads). Further confirming the identity of this band as CD133, its relative abundance was decreased in CaCo-2 cells transfected with CD133-inhibiting siRNAs (lanes 2–4 in Fig 2A).

In contrast, the intensity of unspecific bands (bands indicated by asterisks) was unaffected by CD133 siRNAs). Notably, the detectability of intracellular CD133 in western blot was found to be considerably greater with anti-CD133CT Ab compared to anti-AC133 Ab (Fig 2A, compare CD133 bands in the bottom and middle panels, respectively). This is in clear contrast to the results of flow cytometric evaluations showing greater binding efficacy of anti-AC133 Ab over that of anti-CD133CT Ab (Fig 1B). One reason for this difference may be that AC133 epitope is prevalent in the membrane-associated fraction of CD133 and thus underrepresented in total cell lysates used in western blot analyses (Fig 2A). To address this possibility we compared the detectability of CD133 by anti-AC133 Ab or anti-CD133CT Ab in the plasma membranes (PMs) obtained from CaCo-2 cells by sub-cellular fractionation. To estimate the degree of PMs enrichment, the abundance of Na/K ATPase (constitutive PM resident used as a reference) was compared between PM fractions (“PM”) and unfractionated cell lysates (“input”). In parallel, the abundance of nuclear (histone H1) and cytoplasmic (alpha tubulin) resident proteins was also assessed. The results of the fractionation experiments are shown in Fig 2B. A marked increase in the Na/K ATPase content in “PM” fractions indicates significant enrichment of PMs after fractionation (compare panels “Input” and “PM”). At the same time, the relative representation of cytoplasmic or nuclear resident proteins is drastically decreased in PMs, as expected, compared to unfractionated lysates. Notably, the abundance of AC133 epitope was found to be considerably higher in PMs than in unfractionated lysates (compare CD133/AC133 bands between “input” and “PM”). Importantly, anti-AC133 Ab and anti-CD133CT Ab display similar binding efficacy as evidenced by comparable abundance of CD133/AC133 and CD133CT bands in PMs (Fig 2B). These results indicate that AC133 epitope is predominantly associated with the plasma membrane and thus explain the discrepancy between the data obtained from whole cell-based assays (Fig 1) and those obtained with unfractionated lysates (Fig 2A).

After having confirmed in a model cell line CaCo-2, which is well characterized with respect to CD133/AC133 [43], that both anti-AC133 and anti-CD133CT antibodies bind CD133 protein specifically and with comparable efficacy we used these antibodies to evaluate the level of CD133 expression in GSCs with unknown status of CD133. CD133/AC133 expression was analyzed in five primary cultures of GSCs (No. 10, 1095, 1063, 1080 and 1051) previously established by our group from GBM specimens and characterized extensively with respect to electrophysiological properties [39] and key properties attributed to GSCs such as self-renewal potential, differentiation capacity, tumor-inducing potential and histomorphologic characteristics typical of GBMs (S1 Fig exemplifies GSC characterization results shown for line No. 10). In addition to primary GSC cultures, a sub-population of stem-like glioma cells G112-SP derived from established glioma cell line G112 [46] was also used in this study. G112-SPs possesses key characteristics of GSCs (S1 File) such as unlimited self-renewal (Figure A in S1 File) and high tumorigenicity (Figure B in S1 File) comparable to primary GSC cultures and manifests segregated expression patterns of membrane-associated CD133 as revealed by ummunofluorescence staining with anti-CD133CT antibody (Figure C in S1 File).

**Fig 3:**
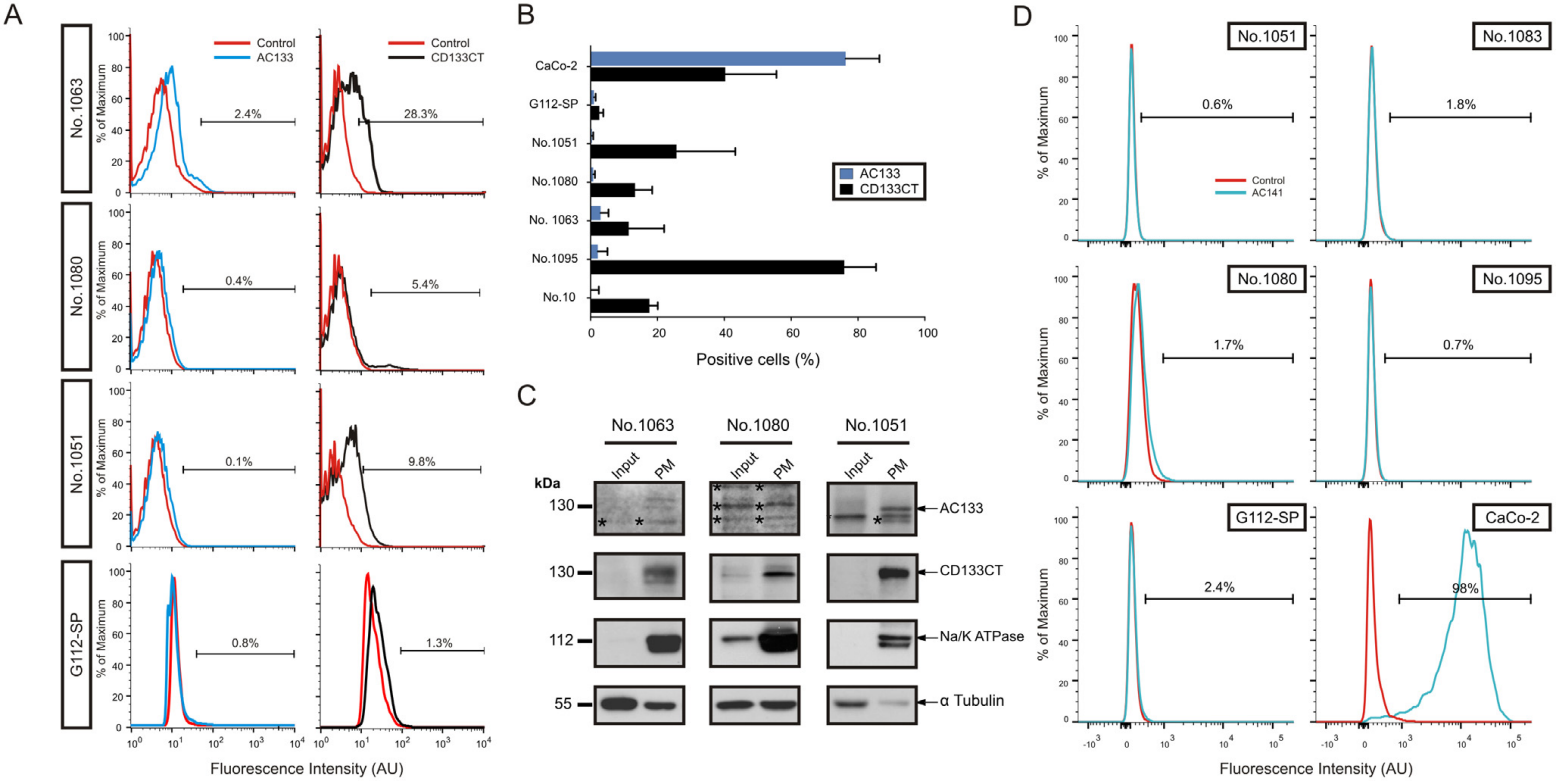
Comparative assessment of the AC133 epitope and CD133 protein in human GCSs. A. Representative histograms showing CD133 surface expression detected by anti-AC133 Ab (*left panels*) or anti-CD133CT Ab (*right panels*) in primary GSCs cultures and stem-like glioma clone G112SP. B. Mean percentage of cells positively labelled with anti-AC133 Ab (*blue*) or anti-CD133CT Ab (*black)* in a panel of GSC lines and CaCo-2 cells used as a positive control. C. Comparative assessment of the total (“input”) and membrane-associated (“PM”) CD133 protein in human GSC lines No. 1063, No. 1080 and No. 1051. 20 μg of proteins were loaded per lane. D. Representative histograms showing surface expression of CD133/2 detected by anti-AC141 Ab in primary cultures of GSCs, stem-like glioma clone G112SP and reference cell line CaCo-2 used as a positive control.

Comparative flow cytometric assessments revealed gross variations between numerical estimates obtained with anti-AC133 or anti-CD133CT antibodies in different GSC lines. In contrast to the abundant expression of the AC133 epitope in CaCo-2 cells (72.75 ± 4.91%, Fig 1B), AC133 estimates in GSCs were found generally low spanning between 1 and 13 percent (Fig 3A, left panels in and Fig 3B, blue bars).

Similarly, extracellular epitope CD133/2 (AC141) that is spatially distinct from AC133 also showed low levels of abundance in our GSCs (Fig 3D). Although the exact mode of recognition of AC133 and CD133/2 epitopes by anti-AC133 or anti-AC141 antibodies, respectively, is currently unknown (rev in [27, 28, 32]), both antibodies have been shown to bind glycosylated structures [30–32]. To test if the lack of GSCs recognition reflects the benerally low abundance of the CD133 protein in our GSCs or is a peculiar feature of anti-AC133 or anti-AC141 antibodies we assessed CD133 expression in GSCs with a glycosylation-independent antibody against CD133CT. Surprisingly, the proportion of CD133+ GSCs detected by anti-CD133CT antibody was considerably higher than that of AC133+ cells in the same cell preparations (Fig 3A, *right panels*, and 3B, *black bars*).

To explain this discrepancy we considered the following possibilities. First, the AC133 epitope may be absent (or inaccessible) in GSCs due to alternative splicing resulting in the N-terminally truncated isoforms of CD133 lacking the region where epitopes AC133 and AC141 are located (aa residues 179–433). We addressed this possibility by analyzing the patterns of normal transcript (called “CD133s2”) and one of the alternatively spliced transcripts (“CD133s1”) coding for an N-terminally truncated isoform of CD133 [42]. As shown in S2 Fig, all GSCs tested do express splice variant CD133s1 lacking a 27 nucleotides-long exon 3 of the CD133 gene [42]. However, there was no apparent correlation between the relative abundance of CD133s1 (S2 Fig) and the binding of anti-AC133 or anti-AC141 antibodies (Fig 3A and 3D). For example, GSCs No. 1063, No. 1080 and G112-SP express CD133s1 at comparable level (S2 Fig) yet differ considerably in the levels of AC133 epitope (Fig 3B). Similarly, GSCs No. 1095 express comparable or even higher amounts of CD133s1 compared to GSCs No. 1080 (S2 Fig) but show lower levels of the AC141 epitope than GSCs No. 1080 (Fig 3D). Notably, all GSCs tested in our study showed considerably lower levels of the CD133 mRNA compared to the normal human brain or CaCo-2 cells (S2 Fig). This finding is somewhat unexpected in light of previous findings that GBM tissues express higher levels of the CD133 mRNA compared to the normal brain tissue [42]. The difference between our results and those by Tabu et al cannot be explained by the use of different primers since the CD133 primers used in our study were the same as those described in the study by Tabu et al [42]. A more likely explanation is that CD133 expression that is thought to be modulated by environmental factors (rev in [26–29]) may vary between GSCs either cultured in vitro or residing in the context of brain tumour tissue.

We next considered the possibility that the AC133 epitope is present in GSCs but poorly accessible to antibodies in the context of undisrupted cell membrane. To address this we assessed the abundance of CD133 protein in the membranes isolated from GSCs. PMs prepared from GSC lines #1051, #1063 and #1080 were analyzed by western blot analysis using anti-CD133CT or anti-AC133 antibodies. The results showed that CD133 protein, while clearly detectable by anti-CD133CT antibody (Fig 3C, panels “CD133CT”), is either unrecognized (line No. 1080) or recognized very weakly (lines No. 1063 and No. 1051) by anti-AC133 antibody (Fig 3C, panels “AC133”). Given that the binding efficacy of AC133 Ab is similar to or even higher than that of CD133CT Ab (Fig 1A, 1B and Fig 2A) these results are consistent with the interpretation that AC133 epitope is either underrepresented or absent in the CD133 protein expressed in GSCs. Of note, there was a considerable degree of cross-reactivity manifested by AC133 Ab, which detects not only CD133 but also other proteins whose mobility does not correspond to either full length CD133 or its known isoforms and which show no characteristic enrichment of CD133 in PMs (Fig 3C, bands marked by asterisks). Based on these results, we conclude that the actual proportion of CD133-expressing GSCs may be considerably higher than predicted by assessments of AC133 or AC141 epitopes. A further argument in support of this conclusion is that all GSCs tested in this study express considerable levels of GSC markers CD15 and CD49f (S3 Fig and Table A in S5 Fig). In accordance with previous studies that have addressed the relationship between AC133 and CD15 [47] or CD49f [48] our results revealed no considerable co-expression of AC133 with either CD15 or CD49f (Table B in S5 Fig).

**Fig 4:**
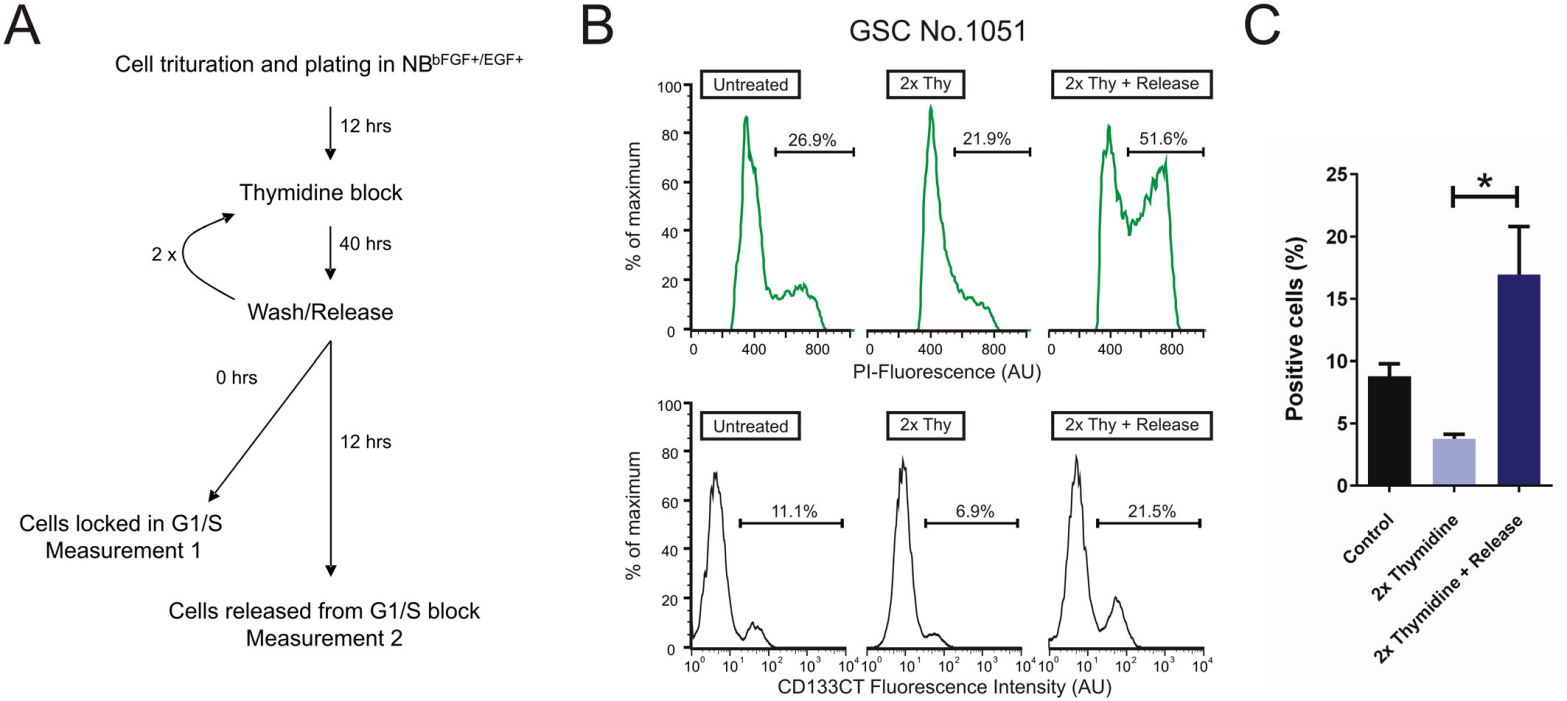
Comparative assessment of surface CD133 in unsynchronized and synchronized GSCs. A. Thymidine treatment scheme used to synchronize human GSCs. B. Representative histograms of No. 1051 GSCs labeled in parallel with propidium iodide (PI, *top row*) and anti-CD133CT Ab (*bottom row*). Fluorescence intensity is represented as arbitrary units (AU) in a linear (PI, *top row*) or logarithmic (anti-CD133CT Ab, *bottom row*) scale. C. Quantitative results of three independent experiments. Shown is the percentage of cells positively labeled with anti-CD133CT Ab in untreated (“control”) or thymidine treated GSCs. GSCs were stained either immediately after the second thymidine treatment (“2xThy”) or after a twelve hours release from a double thymidine block (“2xThy + Release”).

It has been reported that CD133 expression fluctuates throughout the cell cycle in neural stem cells [49], embryonic stem cells and some types of cancer stem cells [50]. To test if such fluctuations also occur in our GSCs, we utilized a synchronization approach to obtain cell populations enriched for specific stages of the cell cycle. GSCs were subjected to a double-thymidine (2xThy) treatment, which enables to enrich cells at the G1/S-phase border of the cell cycle [51]. Surface expression of CD133 was determined by flow cytometry using anti-CD133CT Ab, either immediately after the 2xThy block (measurement 1) or following a 12 hrs release (measurement 2), as depicted in Fig 4A.

In parallel with CD133 staining, cells were stained by propidium iodide (PI) to determine the DNA content (Fig 4B). Synchronization experiments were repeated twice with three replicas in each experiment. The results are summarized in Fig 4C. Consistently with the ability of thymidine to inhibit DNA replication and block G1/S progression [52], treatment with 2xThy led to an inhibition of DNA synthesis as evident from the reduction of the S/G2 fraction (Fig 4B, upper panels). Such a reduction was paralleled by profound (~ 50%) decrease in the percentage of CD133 expressing cells (Fig 4B, lower panel and Fig 4C). After a 12 hours release from the 2xThy block, the number of cells in S/G2 increased (from 21.9% to 51.6%, Fig 4B upper panel) reflecting reactivation of DNA synthesis. In parallel, CD133 expression increased more than 300% (from 6% to 21.5%, Fig 4B lower panel). These results demonstrate that the level of CD133 is not constant but fluctuates during the cell cycle in GSCs (Fig 4C). Further evidence in support of this conclusion comes from our observation that CD133 expressing GSCs often manifest nuclear morphology characteristic of mitotic cells (Figure C in S1 File and S4 Fig). To estimate the proportion of CD133 expressing cells in the mitotic cell population we undertook an observational approach based on microscopic analyses of the morphology of DAPI-stained nuclei. CD133 expressing cells were analyzed with respect to nuclear morphology in two GSC lines (No. 1095 and No. 1080) having a high proliferative potential as determined by the BrdU incorporation assay (data not shown). The results showed that in both lines more than half of CD133 expressing cells exhibit the characteristic nuclear morphology of mitotic cells (S1 Table). Altogether, our findings support the general idea that CD133 is a marker of specific stages of the cell cycle (S, G2 or M) in normal and cancer stem cells [49]. Although the precise mechanism behind cell cycle-dependent variations in CD133 levels is currently unknown our results suggest a previously unconsidered explanation for a direct correlation between CD133 expression and capacity to promote tumour growth [3, 4, 6]. Selection of GSCs with the high level of surface CD133 would enrich for the S/G2/M fraction of cells (Fig 4C) that are ready to enter the mitotic phase and therefore have a high probability of promoting tumour growth. However, the direct relationship between CD133 and tumorigenicity may remain obscured when immunonegativity for AC133 is interpreted as a lack of the CD133 protein. In such a case GSCs that express CD133 but lack immunopositivity for AC133 may be falsely classified as CD133 negative. Although the reason for AC133 non-immunoreactivity in CD133-expressing GSCs is currently unknown the abnormal glycosylation of CD133 in GSCs is likely to be one of the factors involved considering that AC133 reactivity is thought to be influenced by the glycosylation status of CD133 [53, 54] and that aberrant glycosylation is a hallmark of malignancy [55]. Indeed, there is evidence that glycosylation status of the CD133 protein is one (but not the only) essential factor determining the binding of anti-AC133 antibody to the surface CD133 [32, 56, 57] [33], [34]. It has been shown that differentiation in colon carcinoma stem cells is accompanied by loss of the AC133 epitope (but not CD133 protein) presumably due to a change in the tertiary structure of the CD133 protein during differentiation [33]. Our finding that AC133 epitope (but not CD133 protein) is underrepresented in undifferentiated GSCs suggests that loss or masking of the AC133 epitope may also occur independently of differentiation. In addition, in line with previous concerns about technical limitations of AC133-based approaches for evaluating CD133 expression [18], our data underscore the importance of stringent controls to rule out possible impacts of unspecific binding by AC133 Ab. While the methodological and biological reasons for lack of AC133 immunoreactivity in undifferentiated GSCs remain to be identified, clinical implications based on AC133 immunoreactivity need careful consideration.

## Conclusions

This study describes the first identification of GSCs that do express CD133 protein but lack immunoreactivity for AC133. It is shown that AC133 immunoreactivity not always reflects adequately the actual level of CD133 protein expressed in glioma cells. Furthermore, it is also shown that the levels of surface CD133 fluctuate throughout the cell cycle in GSCs with the highest level of CD133 found in S/G2/M. Collectively, our results provide a unifying explanation for existing controversies regarding the association between the tumorigenic potential of glioma cells and CD133+ phenotype and more generally, the potential significance of CD133/AC133 as a predictive biomarker and prognostic indicator of clinical outcome in patients with GBM. On the one hand, the finding that surface expression of CD133 is higher in dividing GSCs explains why CD133+ phenotype is positively associated with the tumour growth promoting ability. On the other hand, such an association may remain unrevealed in a subset of CD133+ GSCs that may have been mistakingly classified as CD133- based on their lack of AC133 immunoreactivity. We conclude that GSCs classifications based exclusively on AC133 immunopositivity may be misleading and result in erroneous classification of CD133+ GSCs as CD133- GSCs and suggest that biological functions and clinical significance of the CD133 protein and AC133 epitope in glioma cells need to be considered independently from each other. Considering that AC133 immunoreactivity is regarded as an indicator of CD133 expression and potential prognostic and predictive marker in GBMs our findings have important clinical implications and support the general idea that CD133 expression is a marker of certain stages of GSCs division rather than constitutive marker of GSCs.

## Supporting Information

S1 Fig Characterization of human GSCs with respect to key cancer stem cell properties. Exemplification of the approach used to characterise different GSC lines used in the study. Shown is the dataset for GSC line No. 10. **A.** Formation of clonal gliomaspheres under serum-free culture condition. **B.** Evaluation of the self-renewal capacity by the limiting dilution assay. Linear regression curve shows the frequency of self-renewing cells in GSC line No. 10. **C.** Phenotypic differentiation of GSCs *in vitro*. In the presence of bFGF/EGF (*left panels*) self-renewing GSCs show weak and heterogeneous expression of the astrocytic marker GFAP (a) and homogeneous expression of the neural stem cell marker nestin (c). Upon the bFGF/EGF withdrawal (*right panels*), GSCs acquire differentiated morphology and manifest increased expression of GFAP (b) without apparent changes in the expression patterns of nestin (d). Cells were counterstained by DAPI. **D.** Tumorigenic capacity in nude mice. Immunohistochemical staining for nestin in tumour xenografts grown from the GSC line No. 10. Brown-stained cells are nestin expressing human GSCs infiltrating through the mouse brain. Inset shows a tumour-free mouse brain stained for human nestin as a control.
(TIF)

S2 Fig Characterization of CD133 transcripts in GSCs and CaCo-2 cells. RT-PCR analysis of full length CD133 mRNA (total length 2598 bp), 632 bp region spanning positions 813–1445 of the CD133 mRNA (total CD133), normally spliced transcript coding for the N-termini of CD133 (CD133s2, positions 278–458) or alternatively spliced transcript CD133s1 (278–431). NB, human normal brain RNA. Asterisks indicate full length alternatively spliced transcripts expressed in the normal brain.
(TIFF)

S3 Fig Flow cytometric immunophenotyping of GSCs for CD15 and CD49f. Representative scatter plots showing the surface expression of CD15 (*x axis*) and CD49f (*y axis*) in primary GSCs cultures and stem-like glioma clone G112SP. Cyan = Isotype control, Red = CD15/CD49f double stained cells.
(TIFF)

S4 Fig CD133 expression in GSCs with mitotic morphology. Representative images of CD133 expressing GSCs (No. 1095 and No. 1080) stained with anti-CD133CT antibody (*red*). Counterstaining by DAPI (*blue*). Magnification 40x.
(TIFF)

S5 Fig Stem cell frequency and relative expression of AC133, AC141, CD15 and CD49f in GSCs. **Table A.** Stem cell frequency determined by limited dilution analysis (Extreme Limited Dilution Analysis, ELDA) [58] and flow cytometric immunophenotyping of primary GSC cultures and G112SP clone. SCF, stem cell frequency. **Table B.** Co-expression of AC133/CD15 and AC133/CD49f in GSCs No. 1051, No.1080, No. 1095 and G112SP evaluated by flow cytometry. “-”= Experiment not performed. All data represent either single experiments or mean ± SEM.
(DOCX)

S1 File Characterization of stem-like clone G112SP with respect to GSC properties. G112SP clone was isolated from the conventional serum-grown glioma cell line G112 [46] by selecting cells capable of gliomasphere formation under serum-free condition. **Figure A.** Representative images of clonal gliomaspheres grown under serum-free culture condition and evaluation of the self-renewal capacity by the limiting dilution assay. The graph shows an example of linear regression analysis used to determine the frequency of self-renewing cells in G112SP clone. **Figure B.** Sections of G112SP xenografts stained with hematoxilin-eosin (HE, magnification: 100x) or anti-human nestin antibody (magnification: 40x). **Figure C.** Conventional immunofluorescence microscopy of G112SP stained with anti-CD133CT antibody either alone (*red*) or a combination of with the cell membrane-selective dye DiO (*green*). Cells were counterstained by DAPI (*blue*). Magnification: 40x.
(TIFF)

S1 Table Relative proportion of CD133 expressing GSCs and cells with mitotic morphology. Numbers correspond to the total number of analyzed cells (“Cell No”), cells exhibiting mitotic morphology (“Mitotic”) and/or stained for CD133.
(DOCX)
