# Supplementary figures and images for: CD133 Expression Is Not Synonymous to Immunoreactivity for AC133 and Fluctuates throughout the Cell Cycle in Glioma Stem-Like Cells
Source: PLoS One. 2015 Jun 18;10(6):e0130519. doi: 10.1371/journal.pone.0130519 (PMC4472699; doi:10.1371/journal.pone.0130519)

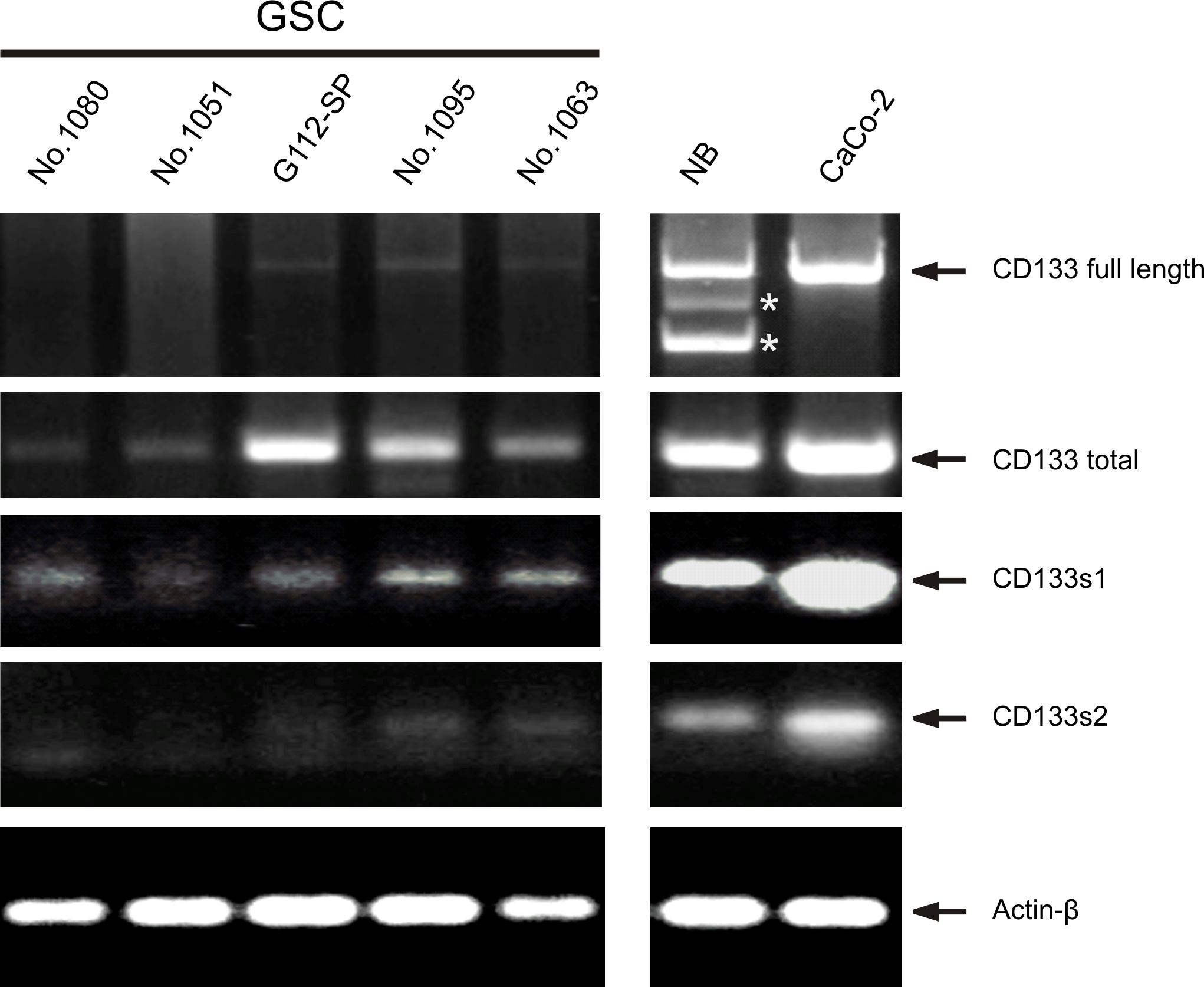

Supplement: S2 Fig — RT-PCR analysis of full length CD133 mRNA (total length 2598 bp), 632 bp region spanning positions 813–1445 of the CD133 mRNA (total CD133), normally spliced transcript coding for the N-termini of CD133 (CD133s2, positions 278–458) or alternatively spliced transcript CD133s1 (278–431). NB, human normal brain RNA. Asterisks indicate full length alternatively spliced transcripts expressed in the normal brain. (TIFF) [file pone.0130519.s002.tiff]

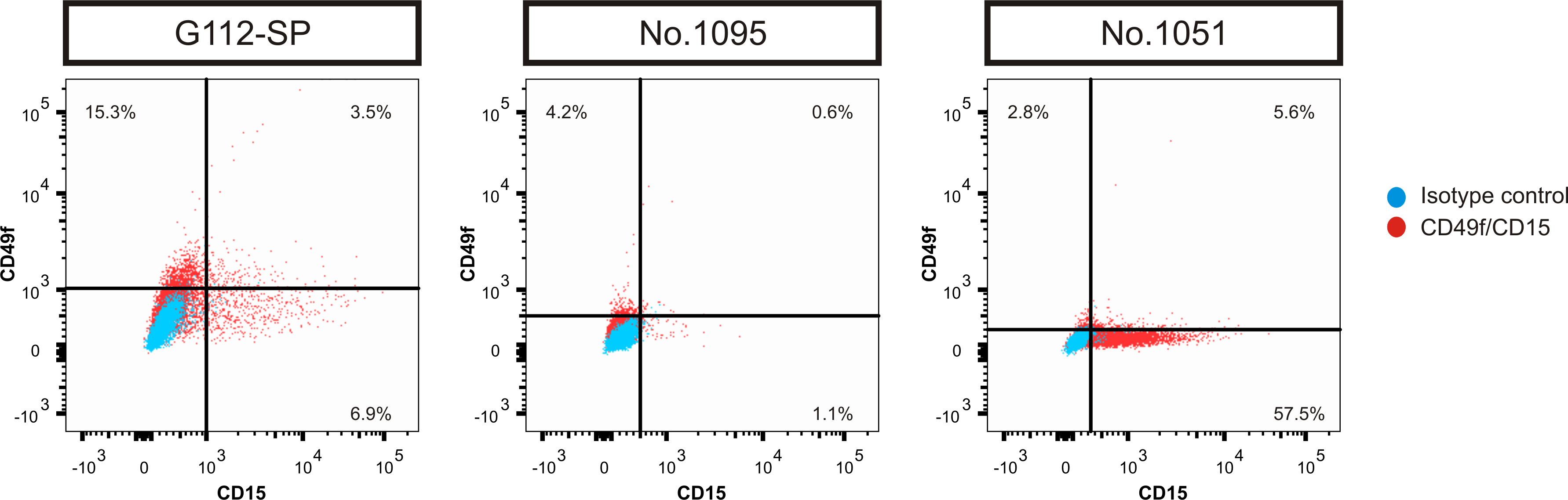

Supplement: S3 Fig — Representative scatter plots showing the surface expression of CD15 (x axis) and CD49f (y axis) in primary GSCs cultures and stem-like glioma clone G112SP. Cyan = Isotype control, Red = CD15/CD49f double stained cells. (TIFF) [file pone.0130519.s003.tiff]

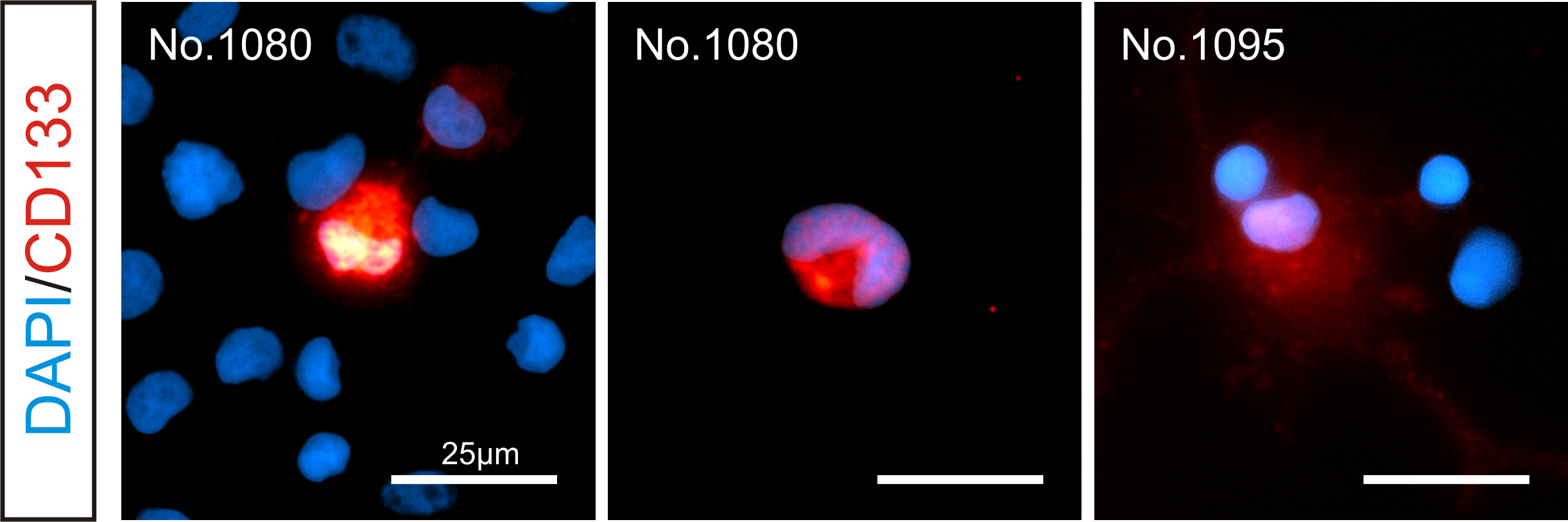

Supplement: S4 Fig — Representative images of CD133 expressing GSCs (No. 1095 and No. 1080) stained with anti-CD133CT antibody (red). Counterstaining by DAPI (blue). Magnification 40x. (TIFF) [file pone.0130519.s004.tiff]

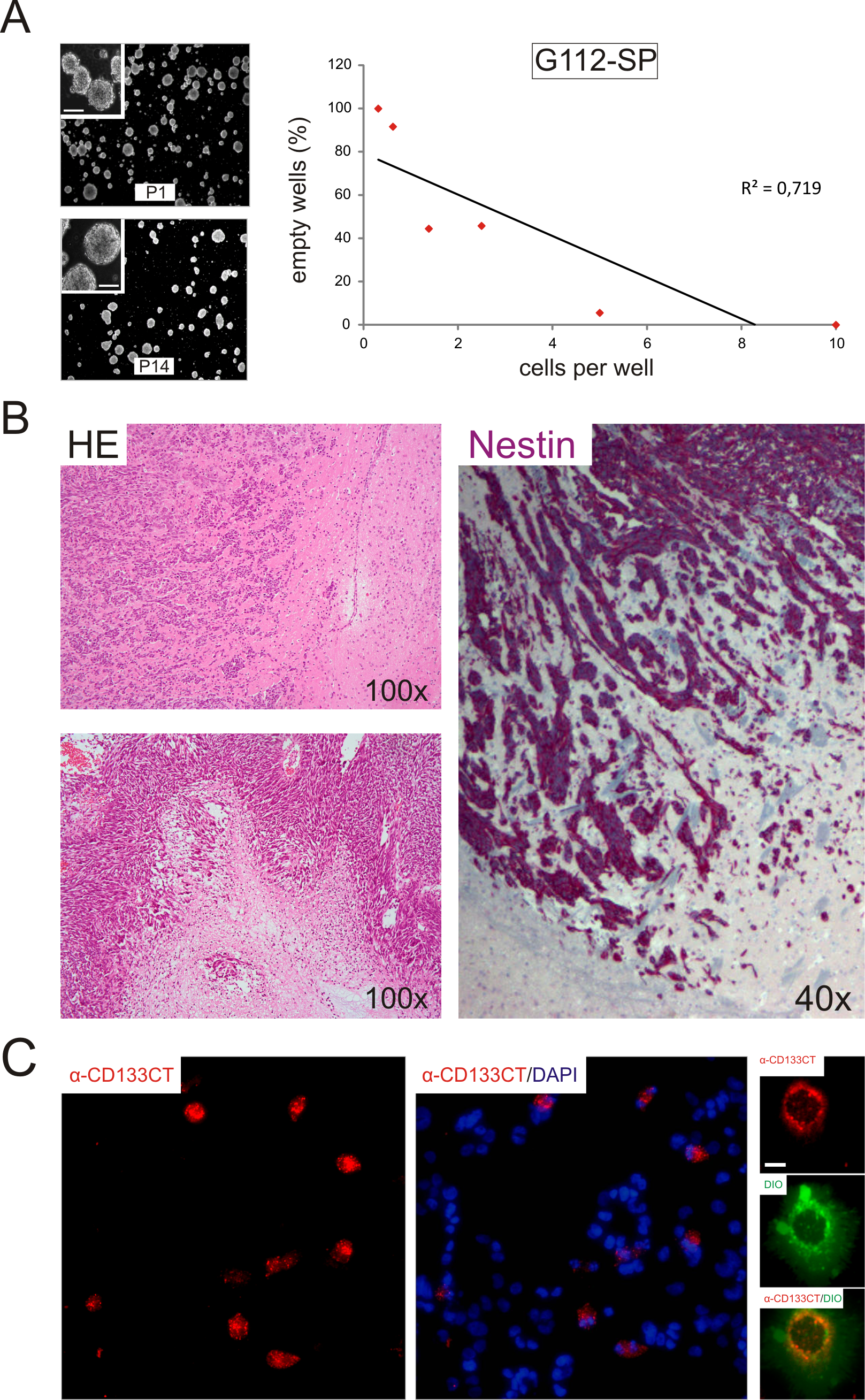

Supplement: S1 File — G112SP clone was isolated from the conventional serum-grown glioma cell line G112 [46] by selecting cells capable of gliomasphere formation under serum-free condition. Figure A. Representative images of clonal gliomaspheres grown under serum-free culture condition and evaluation of the self-renewal capacity by the limiting dilution assay. The graph shows an example of linear regression analysis used to determine the frequency of self-renewing cells in G112SP clone. Figure B. Sections of G112SP xenografts stained with hematoxilin-eosin (HE, magnification: 100x) or anti-human nestin antibody (magnification: 40x). Figure C. Conventional immunofluorescence microscopy of G112SP stained with anti-CD133CT antibody either alone (red) or a combination of with the cell membrane-selective dye DiO (green). Cells were counterstained by DAPI (blue). Magnification: 40x. (TIFF) [file pone.0130519.s006.tiff]
