# Supplementary material for: CD133 Expression Is Not Synonymous to Immunoreactivity for AC133 and Fluctuates throughout the Cell Cycle in Glioma Stem-Like Cells
Source: PLoS One. 2015 Jun 18;10(6):e0130519. doi: 10.1371/journal.pone.0130519 (PMC4472699; doi:10.1371/journal.pone.0130519)
Supplement: S5 Fig — Table A. Stem cell frequency determined by limited dilution analysis (Extreme Limited Dilution Analysis, ELDA) [58] and flow cytometric immunophenotyping of primary GSC cultures and G112SP clone. SCF, stem cell frequency. Table B. Co-expression of AC133/CD15 and AC133/CD49f in GSCs No. 1051, No.1080, No. 1095 and G112SP evaluated by flow cytometry. “-”= Experiment not performed. All data represent either single experiments or mean ± SEM. (DOCX) [file pone.0130519.s005.docx]

|  | **SCF** | **CD15** | **CD49f** | **AC133** | **AC141** |
| --- | --- | --- | --- | --- | --- |
| *G112-SP* | 2.33 | 10.6±1.7 | 15.7±4.0 | 1±0.5 | 5.1±3 |
| *No. 1095* | 5.33 | 1.6±0.2 | 5.8±1.3 | 1±0.1 | 0.7 |
| *No. 1080* | 5.55 | 4.5±1.2 | 31.6 | 1.11 | 6.7±3.5 |
| *No. 10* | 5.59 | 0.3 | 29.5 | 0.1±2.3 | 0.7±0.1 |
| *No.1051* | 5.85 | 51.8±4.7 | 12.36±5.7 | 0.8±0.6 | 3.5±2 |
| *No. 1083* | 15.9 | 0.8±0.2 | Na | 4.36 | 17.7±8.7 |

A

|  | **CD15+/AC133+** | **CD49+/AC133+** | **CD15+/AC141** |
| --- | --- | --- | --- |
| *G112-SP* | 1.4±0.4 | 2.1 ± 0.1 | 2.0 |
| *No. 1095* | 0.3 | 2.18 | - |
| *No. 1080* | 0.1 | - | 3.1 |
| *No.1051* | 0.04±0.03 | 0 | 0.2 |

B
