# Supplementary material for: CD133 Expression Is Not Synonymous to Immunoreactivity for AC133 and Fluctuates throughout the Cell Cycle in Glioma Stem-Like Cells
Source: PLoS One. 2015 Jun 18;10(6):e0130519. doi: 10.1371/journal.pone.0130519 (PMC4472699; doi:10.1371/journal.pone.0130519)
Supplement: S1 Table — Numbers correspond to the total number of analyzed cells (“Cell No”), cells exhibiting mitotic morphology (“Mitotic”) and/or stained for CD133. (DOCX) [file pone.0130519.s007.docx]

|  | **Cell No** | **Mitotic** | **CD133+** | **Mitotic/CD133+** |
| --- | --- | --- | --- | --- |
| *No. 1095* | 109 | 46 | 39 | 23 |
| *No. 1080* | 81 | 39 | 65 | 30 |
